# Classification of acute retinal pigmentepithelitis

**DOI:** 10.1186/s12348-025-00491-9

**Published:** 2025-03-25

**Authors:** Daniel Ahmed-Balestra, Martin Stattin, Katharina Krepler, Siamak Ansari-Shahrezaei

**Affiliations:** 1 Present Address: Karl Landsteiner Institute for Retinal Research and Imaging, Juchgasse 25, Vienna, 1030 Austria; 2 Department of Ophthalmology, Clinic Landstraße, Vienna Health Care Group, Juchgasse 25, Vienna, 1030 Austria; 3 Medical University of Innsbruck, Anichstraße 35, Innsbruck, 6020 Austria; 4 Medical School, Sigmund Freud University Vienna, Campus Prater Freudplatz 3, Vienna, 1020 Austria

## Abstract

**Purpose:**

To present a pediatric case of acute retinal pigment epitheliitis (ARPE) and propose a classification based on imaging findings and prognosis.

**Methods:**

A case report with literature review.

**Results:**

The case demonstrated hallmark ARPE features alongside an atypical disease course, indicating a broader clinical spectrum. Multimodal imaging plays a crucial role in differentiating ARPE from mimicking retinal disorders.

**Conclusion:**

ARPE may represent a spectrum of subtypes with varying prognostic implications. A classification based on age, laterality and imaging biomarkers could improve diagnostic accuracy and patient management.

## Introduction

Acute retinal pigment epitheliitis (ARPE) was first described by Krill and Deutman in 1972 [1]. ARPE is a rarely diagnosed unilateral or bilateral retinal disease that commonly affects young adults, presenting with sudden-onset central or paracentral scotomas.

Multimodal imaging plays a crucial role in the diagnosis of ARPE [2–4]. Optical coherence tomography (OCT) characteristically demonstrates a disruption at the level of the ellipsoid zone, respectively the retinal pigment epithelium (RPE) with focal hyperreflectivity, often extending to the outer nuclear layer. Both, fluorescein angiography (FA) and indocyanine green angiography (ICGA) are essential in the evaluation of ARPE. FA typically reveals transmission hyperfluorescence, while ICGA shows the pathognomonic sign of late halo-like or focal hyperfluorescence. These imaging modalities collectively aid to differentiate ARPE from other retinal disorders.

There is no established treatment for ARPE, as the condition is typically self-limiting, with spontaneous visual recovery expected within a few weeks. However, cases with persistent interdigitation zone loss in structural OCT B-scans and incomplete visual recovery have also been published in the existing literature [2, 3, 5]. Herein we report one young patient with ARPE to discuss the evidence of the disease existence as well as potential ARPE subtypes.

## Case report

**Fig. 1:**
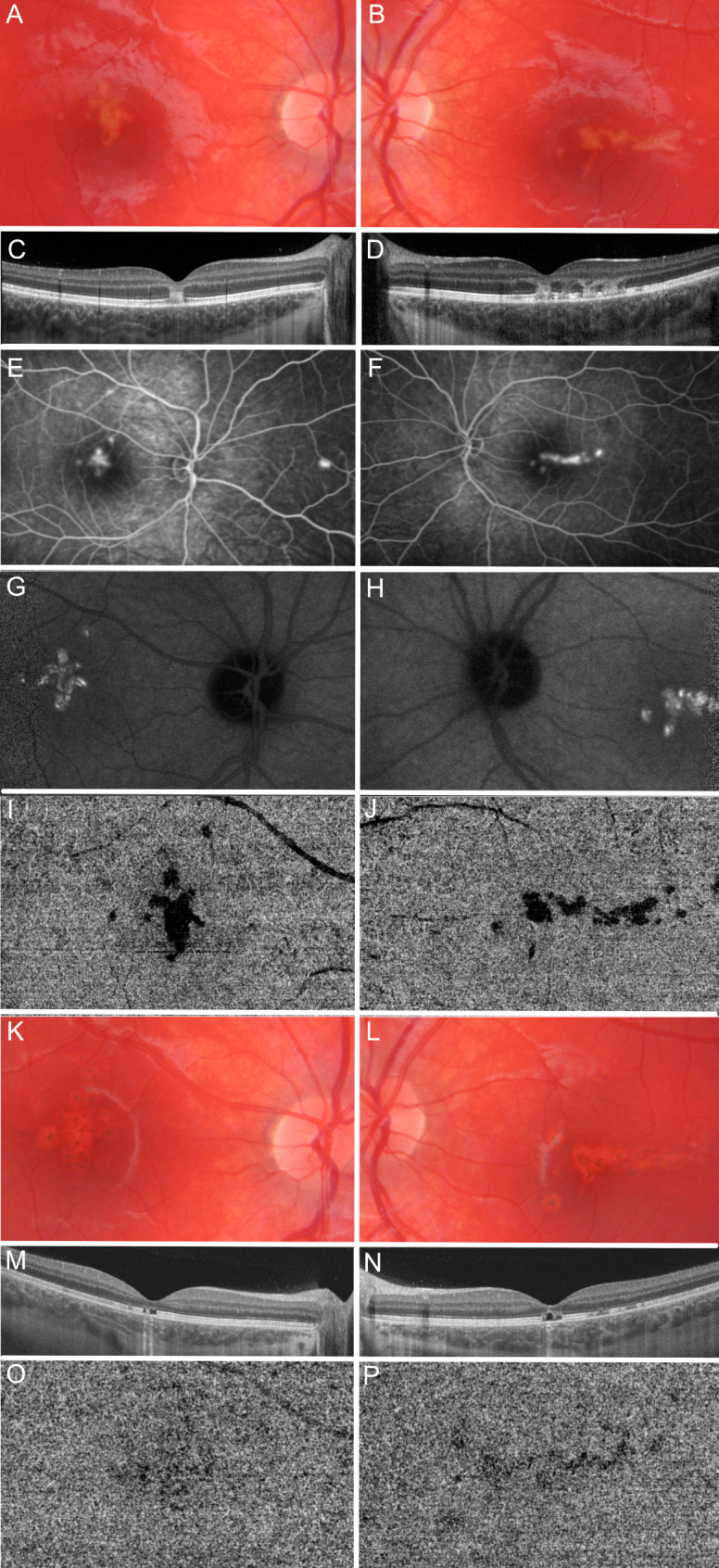
Multimodal imaging of a case of acute retinal pigment epitheliitis type 2

A 10-year-old Caucasian male presented to our tertiary eye care center (Clinic Landstraße, Vienna, medical retina unit) reporting a sudden onset of visual disturbance in both eyes over a few hours. Approximately 10 days prior, he experienced dizziness and nausea with vomiting, for which he received Ibuprofen 400 mg from his parents. Notably, there were no underlying illnesses or long-term medications in his medical history. Upon examination, the best-corrected visual acuity (BCVA) measured 20/50 Snellen (SN) in the right eye (RE) and 20/40 SN in the left eye (LE). The anterior segment appeared normal in both eyes. Fundoscopy revealed grey/brown spots encircled by a yellowish halo over the macula in both eyes, as illustrated in Fig. 1A and B. Swept source (SS)-OCT B-scans demonstrated hyperreflective lesions in both eyes, extending from the RPE to the outer plexiform layer (OPL) (Fig. 1C and D). Corresponding to these lesions, FA revealed transmission hyperfluorescence (Fig. 1. E and F), while ICGA exhibited early blockage followed by the pathognomonic ARPE sign of late hypercyanescence (Fig. 1G and H). Peripheral involvement was noted in the RE (Fig. 1E). Utilizing swept-source SS-OCT angiography (SS-OCTA), we identified a choriocapillaris (CC) dropout in both eyes (Fig. 1I and J). No treatment was initiated based on the presumptive diagnosis of ARPE and a lack of evidence regarding therapeutic options.

Two-weeks follow-up revealed a further BCVA decline to 20/63 SN in the RE, 20/50 SN in the LE and pigment clumping on fundus examination (Fig. 1K and L). SS-OCT showed compact, well-defined hyperreflective lesions and loss of inner segment (IS) and outer segment (OS) substance (Fig. 1M and N). Interestingly, SS-OCTA demonstrated a reduction in CC dropout compared to the baseline (Fig. 1O and P). The patient already underwent treatment with oral prednisolone at a dosage of 1.5 mg/kg/day via a second opinion consultation, which was continued. However, after additional 4 weeks and tapering of prednisolone, the patient’s parents discontinued the medication. Over the ensuing two-year follow-up period, no significant improvement in visual acuity was discernible, culminating in a final BCVA of 20/50 SN in both eyes. Complete resolution of the SS-OCTA CC dropout was observed and a reduction in pigment clumping as indicated by fundoscopy. However, SS-OCT revealed the presence of persistent subfoveal hyperreflective material and demonstrated IS/OS substance loss in both eyes.

## Discussion

The existence of ARPE as a distinct clinical entity was recently questioned in a review article by revisiting the clinical data and images of 86 published ARPE cases [4]. According to the authors, a significant majority (90%) of the reported ARPE cases are more likely attributable to other retinal diseases, with multiple evanescent white dot syndrome (MEWDS) being the most common condition mimicking ARPE. Furthermore, the published commentary “Acute retinal pigment epitheliitis, a diagnostic myth?” suggests that the macular changes observed in ARPE cases might be better interpreted within the spectrum of central serous chorioretinopathy (CSC)/pachychoroid disease, MEWDS or acute macular neuroretinopathy (AMN) [6]. 

We agree with the authors’ opinion that certain retinal diseases have been mislabeled as ARPE. In our case, the modern retinal imaging methodology is characterized by critical distinct biomarkers: a dome-shaped hyperreflective lesion in the interdigitation and ellipsoid zones potentially extending to the outer retinal layers in OCT corresponding to focal late hypercyanescence in ICGA. These phenotypic features have been previously described in ARPE or refer to an unknown retinal disease, as they cannot be assigned to MEWDS, the pachychoroid disease spectrum, retinal light damage or AMN. OCT serves as a pivotal role in the ongoing monitoring of ARPE patients; it may prove insufficient to definitely rule out all disease entities resembling ARPE during the initial presentation. However, high resolution SS-OCT B-scans revealed a hyperreflective lesion reaching from the RPE or interdigitation zone to the OPL. This presentation is atypical for ARPE, as the lesion usually extends to the outer nuclear layer (ONL). Bilateral manifestation and occurrence in minors are also rare aspects of ARPE. To the best of our knowledge, ten cases of bilateral ARPE in minors and young adults have been reported in the literature, with only three documented using multimodal imaging, including ICGA [1, 3, 5, 7–13]. The characteristic hyperfluorescent halo in late ICGA was observed in two of these patients, while intense focal hyperfluorescence in late ICGA was reported in a 9-year-old male, closely resembling our case without visual recovery [5, 11, 13]. Recently, adaptive optics flood illumination ophthalmoscopy imaging revealed persistently reduced parafoveal cone density in ARPE patients compared to healthy controls [13]. Furthermore, cases of unilateral ARPE in young adults without abnormalities in ICGA and interdigitation zone disruption or subtle involvement of the ONL in OCT as reported in “Acute retinal pigment epitheliitis is not a diagnostic myth” might represent a mild ARPE phenotype with self-limiting disease course [2, 3, 14]. In the light of above, we propose an ARPE classification with prognostic capabilities based on age, laterality, OCT and ICGA findings as followed:

- **ARPE type 1**. Predominantly in younger adults with unilateral appearance. Interdigitation zone disruption and possible hyperreflective lesion extension to the ONL in OCT [2, 3, 14, 15].
  - Unremarkable ICGA findings and visual restoration within weeks.
  - Focal or halo-like hyperfluorescence in late ICGA. Visual restoration within weeks, rarely persistent.
- **ARPE type 2**. Predominantly in minors or younger adults with bilateral appearance. Interdigitation zone disruption and possible hyperreflective lesion extension to the OPL in OCT. Focal or halo-like hyperfluorescence in late ICGA. Persistent BCVA decline possible [5, 11].

The query whether peripheral involvement is a defining characteristic of ARPE type 2, as identified in our patient, requires clarification through additional case reports utilizing widefield-ICGA. Moreover, our research revealed only one more ARPE case report involving a minor, affecting an 11-year-old patient with unilateral involvement [16]. Notably, in all other previously published ARPE cases among minors, bilateral involvement was consistently observed. The rarity of bilateral affection in ARPE cases overall supports our hypothesis that ARPE in minors may represent a distinct subcategory within the broader ARPE spectrum or potentially constitute an independent disease entity. In summary, the accurate diagnosis of ARPE necessitates a multimodal imaging approach, including ICGA. An age-dependent disease expression might explain the heterogenous findings in this orphan disease.

## Data Availability

No datasets were generated or analysed during the current study.
